# Translational control of *Toxoplasma gondii* differentiation

**DOI:** 10.1042/BSR20253672

**Published:** 2026-04-02

**Authors:** Fengrong Wang

**Affiliations:** 1 Department of Biomedical Sciences, Iowa State University, Ames, IA 50010, U.S.A.; 2 Department of Microbiology and Immunology, University of Michigan Medical School, Ann Arbor, MI 48109, U.S.A.; 3 Department of Biological Chemistry, University of Michigan, Ann Arbor, MI 48109, U.S.A.

**Keywords:** Alternative splicing, Cell differentiation, Non-coding RNA, RNA editing, RNA-binding proteins, Toxoplasma gondii, Translation, Translation factors

## Abstract

*Toxoplasma gondii* is a globally prevalent protozoan parasite capable of establishing lifelong infections in its host. While acute infection is often asymptomatic, reactivation of latent bradyzoites can cause severe disease, particularly in immunocompromised individuals. Current therapies are ineffective against chronic infection, underscoring critical gaps in our understanding of bradyzoite biology and the molecular mechanisms governing stage conversion. Recent studies have identified translational control as a central regulator of *T. gondii* differentiation. This review highlights the roles of canonical translation initiation factors (eIF2α, eIF1.2, and eIF4E1), RNA-binding proteins (RBPs; BFD2/ROCY1, Alba1, and Alba2), and RNA modifications (with pseudouridylation representing the best-characterized modification currently linked to differentiation), as well as alternative splicing and non-coding RNAs in shaping stage-specific translational programs. This review further discusses underexplored mechanisms, including non-canonical initiation pathways, upstream open reading frames, transcript-level RNA modifications, ribosome heterogeneity and rRNA modifications, elongation and termination control, uncharacterized RBPs, and post-translational modifications of translation factors, that may coordinate proteome remodeling during differentiation. Together, established translational regulators and these emerging pathways highlight translational control as a central driver of parasite persistence and a promising therapeutic target for chronic toxoplasmosis.

## Introduction to *T. gondii* and stage conversion

**Figure 1:**
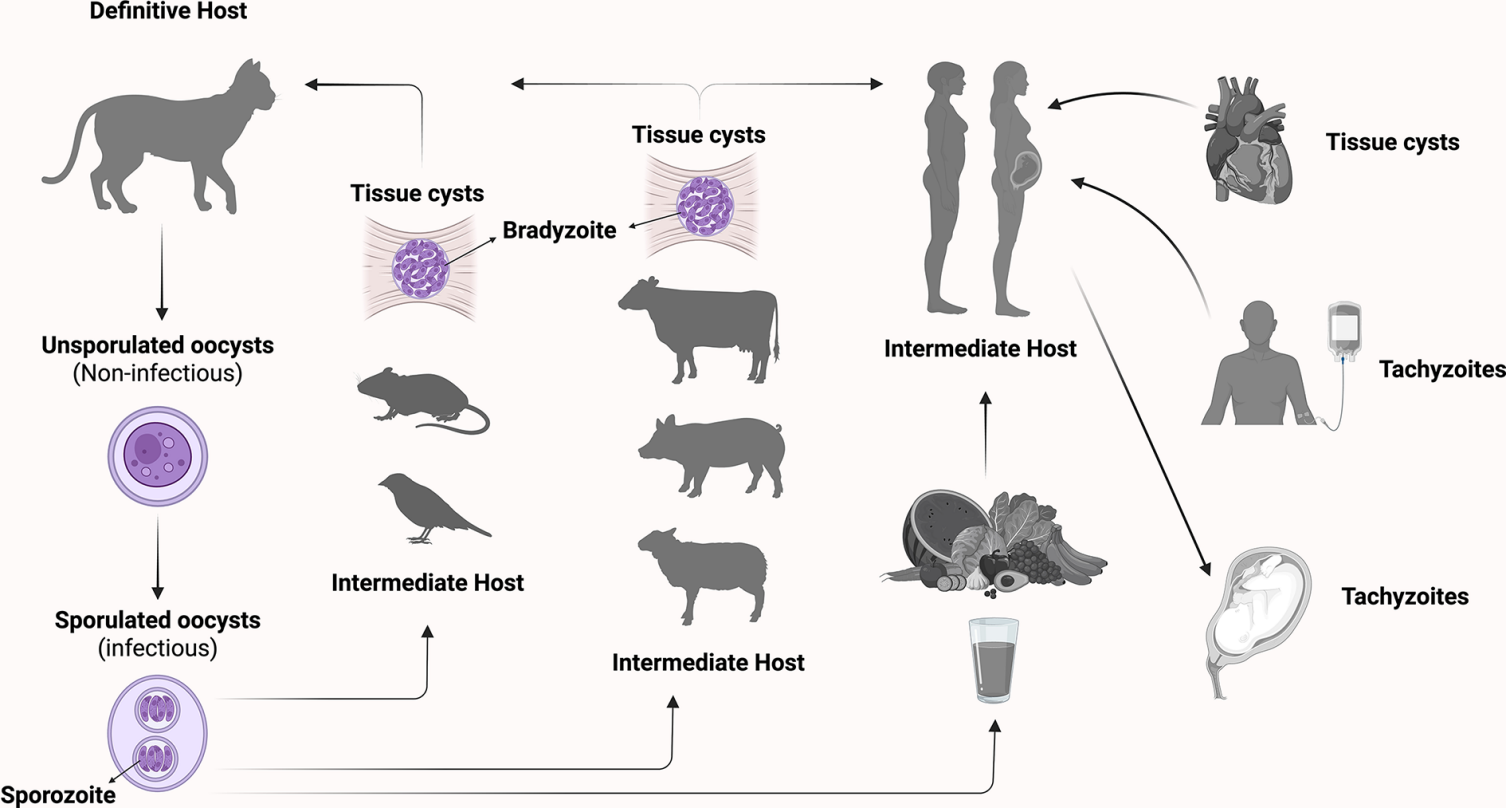
*T. gondii* transmission cycle This schematic illustrates the complex transmission cycle of *T. gondii*, involving both definitive and intermediate hosts. Felids (e.g., domestic cats) are the definitive hosts, and shed unsporulated oocysts in their feces. These oocysts sporulate in the environment and become infectious. Intermediate hosts, including rodents, birds, and livestock, become infected by ingesting sporulated oocysts from contaminated soil, water, or food. Humans may acquire infection through consumption of contaminated water or unwashed fruits or vegetables, or by eating undercooked meat containing tissue cysts. Additional routes of transmission in humans include organ transplantation, blood transfusion, and vertical transmission during pregnancy. Within intermediate hosts, the parasite differentiates from rapidly replicating tachyzoites into slowly dividing bradyzoites that reside within tissue cysts. These cysts primarily localize in the brain, heart, and skeletal muscle, where they can persist for life and may reactivate under conditions of immunosuppression. Adapted from artwork by ©ABCD, Karin de Lange.

*Toxoplasma gondii* is an obligate intracellular parasite renowned for its ability to infect nearly all warm-blooded animals, including humans [1]. As the causative agent of toxoplasmosis, it ranks among the most widespread zoonotic pathogens globally, with human seroprevalence rates ranging from 16% to over 60%, influenced by geographic, socioeconomic, and environmental factors [2]. This widespread prevalence is driven by a biphasic life cycle that alternates between definitive felid hosts and diverse intermediate hosts (including humans, livestock, and birds), ensuring ecological persistence and transmission (Figure 1).

In definitive felid hosts, sexual reproduction within the intestinal epithelium generates unsporulated oocysts, which are shed into the environment via feline feces [3]. These oocysts undergo sporulation, a process requiring oxygen, humidity, and ambient temperatures, to mature into environmentally resilient, infectious forms containing sporozoites [4]. Intermediate hosts, including humans, become infected through ingestion of sporulated oocysts from contaminated soil, water, or food, or by consuming undercooked meat containing tissue cysts harboring dormant bradyzoites [4,5]. These stages differentiate into rapidly replicating tachyzoites that mediate acute infection and systemic dissemination. Less common routes include organ transplantation, transfusion of blood products containing tachyzoites, and congenital transmission following acute maternal infection [6] (Figure 1).

**Figure 2:**
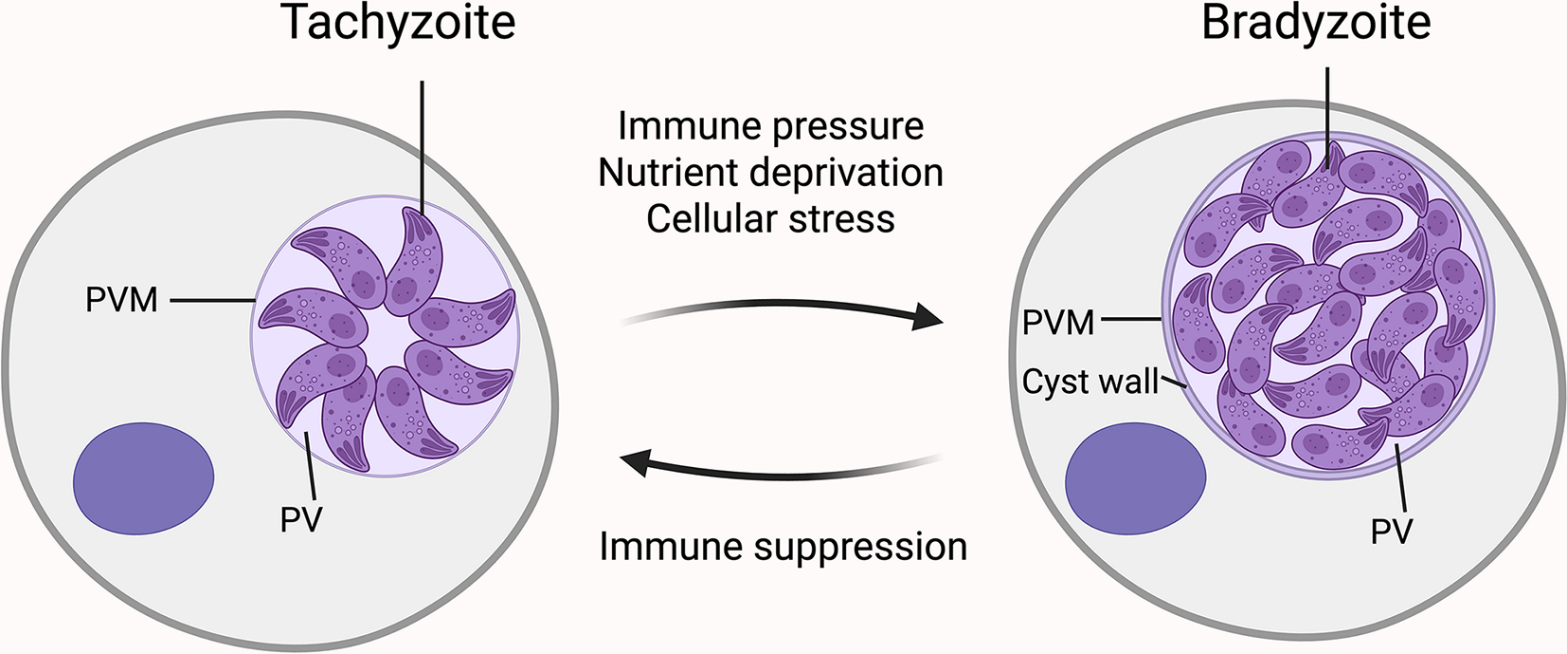
Stage conversion between tachyzoites and bradyzoites in *T. gondii* This diagram illustrates the interconversion between the two major life cycle stages of *T. gondii* within intermediate host cells. Tachyzoites are the rapidly replicating form responsible for acute infection and reside within the parasitophorous vacuole (PV), which is enclosed by the parasitophorous vacuole membrane (PVM). Under stress conditions, including immune pressure, nutrient deprivation, and other cellular stressors, tachyzoites differentiate into bradyzoites, the slow-growing, latent form. Bradyzoites reside within a cyst enclosed by a thick cyst wall that forms within the PVM and are responsible for chronic infection. Immunosuppression can trigger reactivation of bradyzoites into tachyzoites, leading to disease recurrence.

Within intermediate hosts, *T. gondii* transitions between two key developmental stages: tachyzoites, responsible for acute infection, and bradyzoites, which establish chronic latency [7]. Tachyzoites rapidly proliferate within host cells, replicating inside a parasitophorous vacuole (PV) [8]. Their unchecked replication and eventual egress via host cell lysis drive systemic dissemination and tissue damage [9]. While the host immune response clears most tachyzoites, surviving parasites differentiate into bradyzoites, which are slower-growing, metabolically quiescent forms enclosed within a cyst wall [10]. These tissue cysts predominantly localize to immune-privileged sites such as the brain, heart, and skeletal muscles, enabling lifelong persistence [11]. Immunosuppression can reactivate bradyzoites into tachyzoites, triggering recrudescent infection [12] (Figure 2).

Clinically, toxoplasmosis manifests variably depending on host immune status. In immunocompetent individuals, acute infection is often asymptomatic or mild [13,14], whereas immunocompromised patients, such as those with HIV/AIDS, organ transplant recipients, or individuals receiving immunosuppressive therapies, are at high risk of severe, life-threatening complications, including toxoplasmic encephalitis, myocarditis, pneumonitis, and disseminated disease [15–18]. Additionally, primary infection in pregnant women can lead to congenital transmission, resulting in a spectrum of fetal outcomes ranging from asymptomatic infection to severe neurological impairment, chorioretinitis, hydrocephalus, or fetal loss, depending largely on the gestational timing of maternal infection [13,19].

Stage conversion is influenced by external stressors, including nutrient deprivation [20–22], alkaline pH (8.0–8.2) [23], elevated temperatures (e.g., 43°C) [23], metabolic inhibitors [24,25], and immunity or inflammatory signals [24,26,27]. This developmental switch entails extensive cellular remodeling, including the formation of a highly glycosylated cyst wall underneath the PV membrane [11,28–31] (Figure 2), metabolic shifts toward glycolysis [32,33], accumulation of cytoplasmic starch granules [34–37], and activation of stage-specific gene expression programs [38–41]. While transcriptional regulation sets the stage for gene expression and post-transcriptional modifications determine the availability and stability of messenger RNA (mRNA), translational control serves as the final checkpoint governing protein synthesis, enabling rapid, localized, and on-demand production of proteins in direct response to environmental stimuli. Mounting evidence implicates translational regulation as a central mechanism by which *T. gondii* reprograms its proteome to adapt to host stressors, evade immunity, and sustain chronic infection [42–47]. Unraveling these molecular pathways is essential for developing therapies to disrupt persistent toxoplasmosis and its clinical sequelae.

## Introduction to protein translation

**Figure 3:**
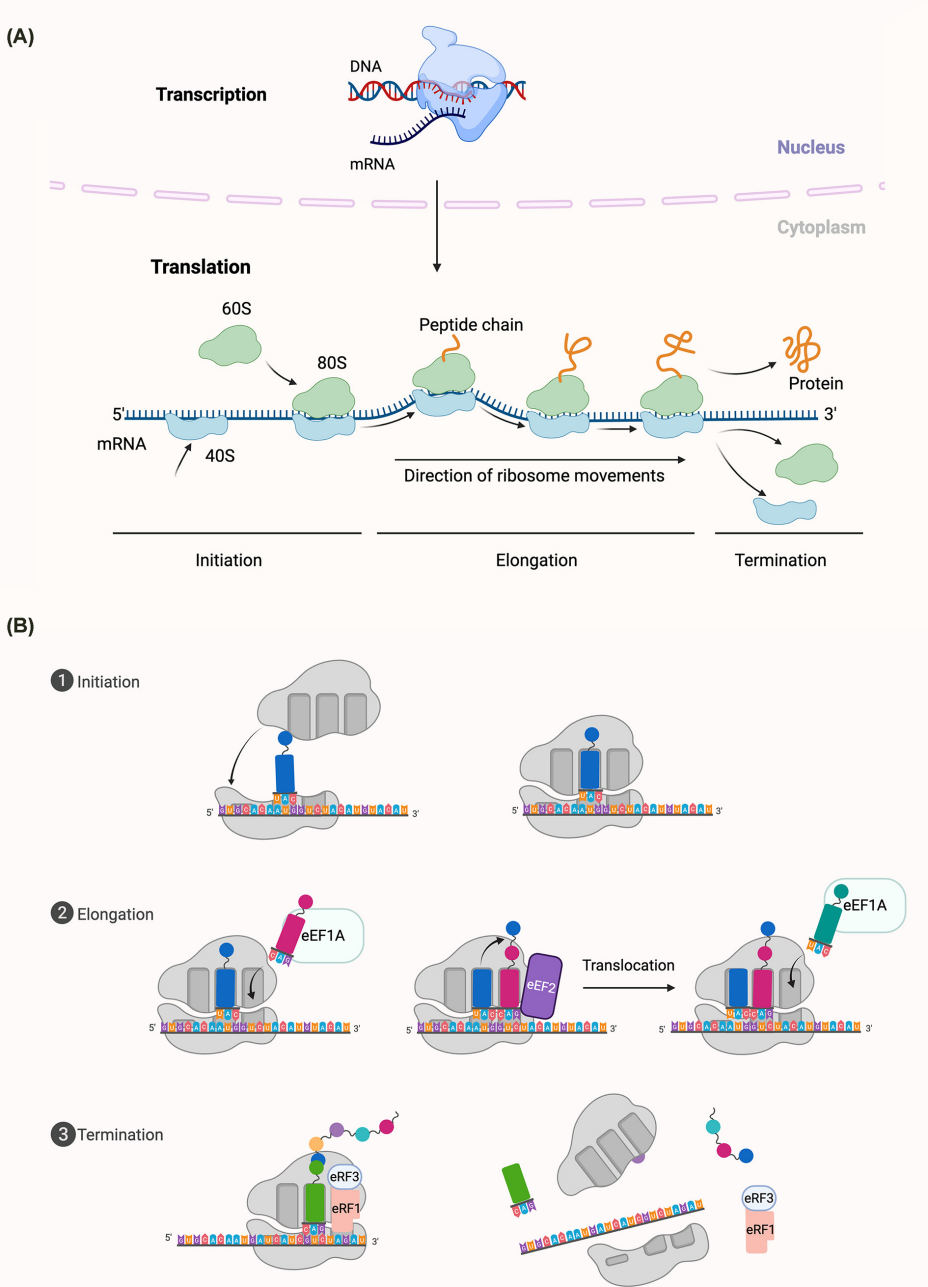
Overview of eukaryotic gene expression: transcription and translation (**A**) This schematic outlines the flow of genetic information from DNA to protein. In the nucleus, DNA is transcribed into mRNA, which is exported to the cytoplasm. Translation occurs in the cytoplasm and proceeds through three main stages: initiation, elongation, and termination. (**B**) During initiation, the 40S ribosomal subunit, together with initiation factors, is recruited to the 5′ end of the mRNA and scans for the start codon. Upon start codon recognition, the 60S subunit joins to form the 80S ribosome, initiating peptide synthesis. Elongation proceeds as the ribosome moves along the mRNA, catalyzing peptide bond formation and elongating the nascent polypeptide chain. Termination occurs when a stop codon is encountered, leading to the release of the completed polypeptide and the disassembly of the ribosome.

Protein translation is the biological process by which ribosomes decode the genetic information carried by mRNA to synthesize functional proteins, representing the final step in the central dogma of molecular biology. This intricate and energetically demanding process is universally conserved across eukaryotes but exhibits specialized adaptations in pathogens like *T. gondii* [45,91], enabling survival under host-induced stresses. Translation occurs in three sequential stages: initiation, elongation, and termination, with each orchestrated by a suite of conserved molecular machinery that ensures fidelity while allowing adaptation to varying cellular conditions (Figure 3).

### Initiation

Translation initiation is the most tightly regulated and rate-limiting phase of protein synthesis. In eukaryotes, including *T. gondii*, initiation begins with the assembly of the 43S pre-initiation complex (43S), which consists of the 40S small ribosomal subunit, several eukaryotic initiation factors (eIFs), such as eIF1, eIF1A, eIF3, and the ternary complex (eIF2 bound to GTP and methionyl initiator transfer RNA, Met-tRNA_i_^Met^) [48,49]. The 43S complex is recruited to the 5′ end of mRNA through interaction with the eIF4F complex, composed of eIF4E (cap-binding protein), eIF4A (an ATP-dependent RNA helicase), and eIF4G (a scaffold protein), forming the 48S initiation complex (48S) [48].

During scanning, the 48S complex translocates along the 5′ untranslated region (UTR) of the mRNA in a 5′-to-3′ direction in an ATP-dependent manner [50,51]. This movement is primarily driven by the RNA helicase activity of eIF4A, which unwinds secondary structures in the 5′-UTR that could otherwise impede ribosomal progression. This helicase activity is enhanced by cofactors such as eIF4B and eIF4H, which stimulate eIF4A’s processivity and RNA-binding capacity [52]. Efficient scanning allows the ribosome to locate the correct start codon, typically an AUG embedded within a favorable Kozak consensus sequence (G/ANNAUGG), characterized by a purine at the -3 position and a guanine at +4 [53]. In *T. gondii*, the preferred sequence consists of an adenine at -3 and guanine at +4 [54].

The fidelity of this scanning process is safeguarded by eIF1 and eIF1A, which stabilize an open conformation of the 48S complex and prevent initiation at near-cognate or out-of-frame codons [55]. Once Met-tRNA_i_^Met^ base-pairs with the start codon, the 48S complex undergoes a conformational shift to a closed state that signals proper codon-anticodon recognition [56]. This transition triggers a cascade of events: eIF1 is released, eIF5 occupies its vacated position near the P site of the ribosome [52,57,58], and eIF5 also functions as a GTPase-activating protein (GAP), stimulating GTP hydrolysis on eIF2 [59,60]. The subsequent release of inorganic phosphate (Pi) from eIF2 [61] commits the complex to the selected start codon and primes it for subunit joining through the recruitment of eIF5B.

**Figure 4:**
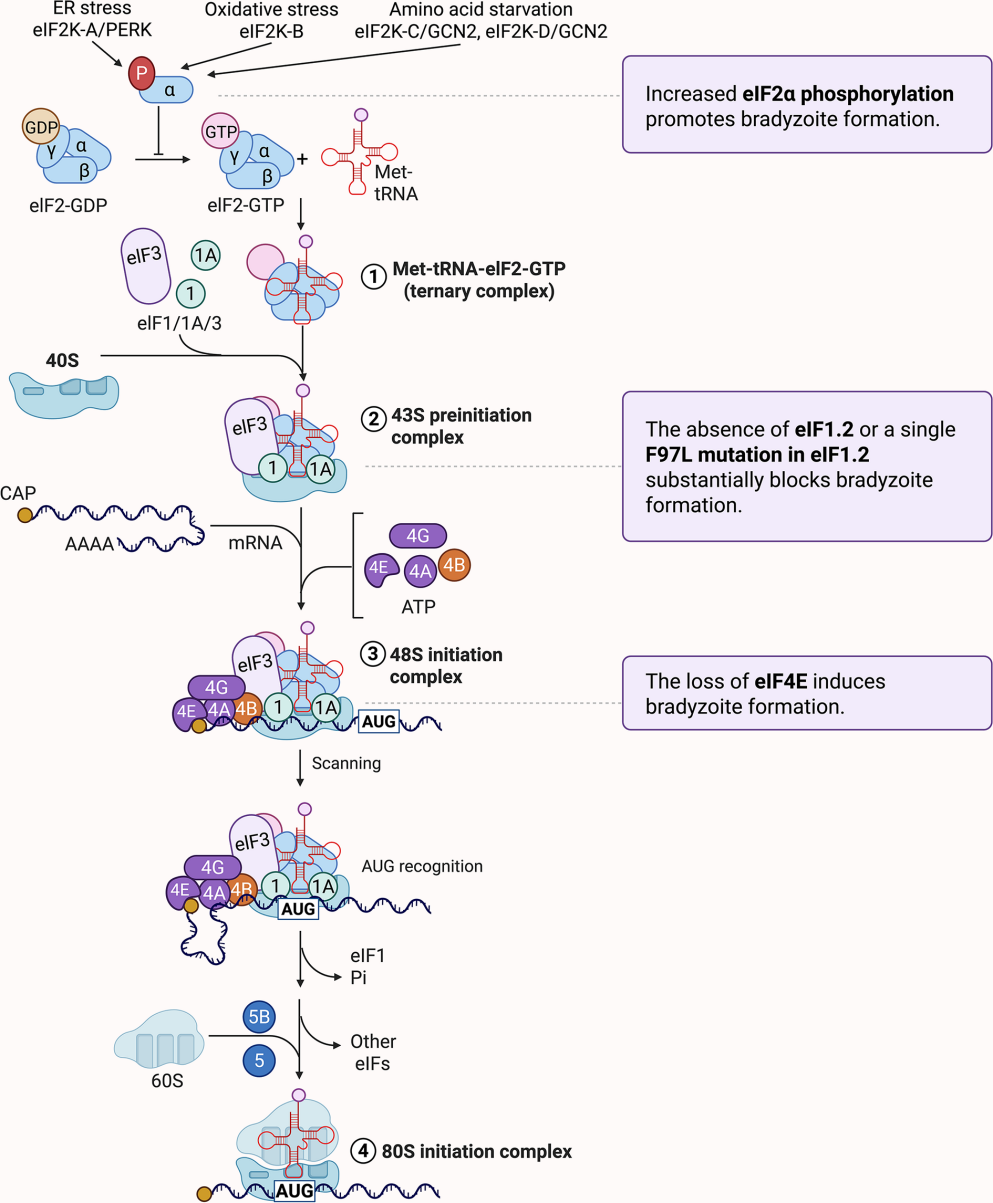
Translational control of *T. gondii* differentiation This diagram illustrates the molecular events of translation initiation and their regulatory roles in *T. gondii* bradyzoite formation. Translation initiation begins when the 40S ribosomal subunit associates with eIF1, eIF1A, and eIF3. The ternary complex (eIF2-GTP-Met-tRNA_i_^Met^) then joins this assembly to form the 43S pre-initiation complex (Steps 1 and 2). In parallel, the eIF4F complex (eIF4E, eIF4G, eIF4A) with cofactors such as eIF4B, binds to the 5′ cap of mRNA and facilitates its recruitment to the 43S complex, forming the 48S initiation complex (Step 3). This complex scans the mRNA for the AUG start codon. Upon AUG recognition, eIF1 is released, and the 60S ribosomal subunit joins to form the functional 80S initiation complex (Step 4). Genetic and environmental perturbations in this pathway impact developmental fate. ER stress (e.g., tunicamycin or calcium ionophore treatment) activates the PERK-like kinase eIF2K-A, and oxidative stress (e.g., sodium arsenite) activates eIF2K-B, as shown by genetic deletion, kinase inhibition, and phospho-eIF2α analyses; in both cases, eIF2α phosphorylation suppresses global translation and promotes bradyzoite differentiation [42,93,94]. The GCN2-like kinases eIF2K-C and eIF2K-D are activated by amino acid starvation or post-egress stress and phosphorylate eIF2α, but their direct role in differentiation remains unclear [95]. Pharmacological stabilization of phosphorylated eIF2α (e.g., salubrinal, guanabenz) induces differentiation. In parallel, depletion of eIF4E1 triggers spontaneous bradyzoite formation [44], whereas deletion or F97L mutation of eIF1.2 impairs start codon selection and blocks differentiation [45,99].

eIF5B, itself a GTPase, stabilizes Met-tRNA_i_^Met^ in the P-site and promotes the joining of the 60S large ribosomal subunit to the 48S complex [62]. This coordinated transition culminates in the formation of the 80S initiation complex, marking the completion of initiation and the transition to the elongation phase of translation (Figures 3 and 4). These molecular checkpoints ensure that translation initiates precisely at the correct codon, thereby preventing aberrant protein synthesis.

In *T. gondii*, although the full molecular details of these events remain to be elucidated, the presence of conserved orthologs of eukaryotic initiation factors, including eIF1, eIF1A, the eIF2 complex, and the eIF4F complex, suggests that the core features of this regulatory framework are maintained, with potential parasite-specific adaptations yet to be characterized.

### Elongation

Elongation is a cyclic process that drives the stepwise addition of amino acids to the growing polypeptide chain [63]. Each cycle begins with the delivery of an aminoacyl-tRNA to the ribosomal A site by the elongation factor eEF1A, which forms a ternary complex with GTP and the charged tRNA [64]. Accurate codon-anticodon pairing is ensured through kinetic proofreading mechanisms before GTP hydrolysis triggers the release of eEF1A and accommodation of the tRNA into the A site [65].

Peptide bond formation is catalyzed by the peptidyl transferase center of the large ribosomal subunit, transferring the nascent polypeptide chain to the amino acid in the A site. Translocation of the ribosome along the mRNA is then facilitated by eEF2 in a GTP-dependent manner, shifting the peptidyl-tRNA from the A site to the P site and clearing the A site for the next incoming tRNA [66].

In *T. gondii*, canonical eukaryotic elongation factors such as eEF1A (aminoacyl-tRNA delivery) and eEF2 (ribosome translocation) are conserved based on sequence homology with other eukaryotes, suggesting the core elongation machinery is maintained. Structural studies in related apicomplexans (e.g., *Plasmodium*) reveal divergent ribosomal proteins and rRNA expansion segments, which could alter elongation factor binding sites or ribosome processivity [67]. In *T. gondii*, the cytoplasmic ribosome may exhibit similar modifications, potentially affecting eEF2 GTPase activity during translocation and the efficiency or fidelity of eEF1A-mediated aminoacyl-tRNA delivery.

### Termination

The termination phase marks the final stage of protein synthesis, occurring when the ribosome encounters one of three stop codons (UAA, UAG, or UGA) [68]. In canonical eukaryotic systems, this process is mediated by two release factors: eRF1, which recognizes all three stop codons, and eRF3, a GTPase that assists eRF1 activity [63,69]. eRF1 structurally mimics a tRNA and binds the ribosomal A site, where its conserved Gly-Gly-Gln (GGQ) motif catalyzes the hydrolysis of the bond between the polypeptide and the tRNA in the P site, releasing the nascent polypeptide chain from the ribosome [70,71]. The GTPase eRF3 associates with eRF1 in a GTP-bound complex and enhances stop codon recognition and peptide release, with subsequent GTP hydrolysis driving release factor dissociation [72,73]. Following peptide release, the ABC-type ATPase ABCE1 plays a critical role in ribosome recycling by splitting ribosomal subunits and resolving post-termination complexes [80,81]. This carefully orchestrated process ensures the efficient release of both the completed protein product and the ribosomal machinery, which is then recycled for subsequent rounds of translation [69] (Figure 3).

The fidelity and efficiency of translation termination are influenced by multiple contextual factors. The nucleotide sequences flanking stop codons, particularly at the +4 position, can significantly modulate termination efficiency, with certain contexts promoting readthrough events [74,75]. Additionally, mRNA secondary structures downstream of stop codons and interactions with the poly(A) tail may affect the timing and success of termination [76,77]. Furthermore, termination is linked to mRNA surveillance mechanisms, such as nonsense-mediated decay (NMD), which detects and degrades mRNAs with premature termination codons to prevent synthesis of aberrant proteins [78,79].

Defects in termination machinery can cause translational readthrough, producing C-terminally extended proteins that are typically harmful but sometimes beneficial, such as the antiangiogenic VEGF-Ax isoform generated by programmed readthrough [82]. Persistent ribosomal stalling at termination sites can also trigger ribosome collisions, which activate ribosome quality control (RQC) pathways, including no-go decay and nonstop decay [83–85]. These pathways are essential for resolving aberrant translation events and maintaining protein homeostasis. Importantly, mutations in human termination factors (e.g., eRF1, eRF3, or UPF1) have been associated with various neurodevelopmental disorders, cancer, and inherited diseases [86–88], underscoring the physiological relevance of precise termination.

While the termination process in *T. gondii* remains poorly characterized, investigating its mechanisms could advance our understanding of translation regulation in apicomplexan parasites and potentially reveal parasite-specific vulnerabilities exploitable for therapeutic interventions.

In *T. gondii*, translation not only produces essential proteins for cellular function and survival but also serves as a critical mechanism for post-transcriptional gene regulation. By selectively translating specific mRNAs, parasites can rapidly respond to environmental cues, direct stage-specific differentiation, and adapt to stress, even in the absence of transcriptional changes [89,90]. This dynamic control of protein synthesis is essential for survival and persistence across diverse host environments.

## Protein translation as a regulatory nexus in *T. gondii* differentiation

As *T. gondii* transitions from the rapidly replicating tachyzoite stage to the latent bradyzoite form under stress, increasing evidence highlights translational control as a critical mechanism for modulating stage-specific gene expression. These regulatory strategies operate at multiple levels, including translation initiation pathways, dynamic RNA-binding proteins (RBPs), and mRNA modifications, which collectively fine-tune protein synthesis essential for differentiation. The major translational regulators identified to date, their molecular functions, and the differentiation phenotypes observed upon genetic or pharmacological perturbation are summarized in Table 1.

**Table 1:** Regulators of translational control during *T. gondii* stage conversion

Regulator	Core function	Perturbation	Differentiation outcome	Key references
eIF2⍺	Global initiation control	Stress-induced phosphorylation; salubrinal/guanabenz (block dephosphorylation)	Promotes differentiation and maintains latency	[42,93–98]
eIF2K-A (PERK-like)	ER stress kinase (PERK-like)	Inhibition (GSK2606414)	Reduces differentiation under ER stress	[42,96]
eIF2K-B	Oxidative stress kinase	Deletion	Impaired cyst formation	[94]
eIF2K-C/eIF2K-D	Starvation-responsive kinases (GCN2-like)	Activation	Role in differentiation unclear	[95]
eIF1.2	Start codon fidelity	Mutation (F97L) or deletion	Blocks differentiation	[45,99]
eIF1.1	eIF1 paralog	Deletion	No effect on differentiation	[99]
eIF4E1	Cap-dependent translation initiation	Conditional knockdown (mAID) or inhibition (4EGI-1)	Induces spontaneous differentiation	[44]
BFD2/ROCY1	Activates BFD1 translation	Deletion or zinc-finger mutation	Blocks differentiation	[46,103]
Alba1	3′-UTR-mediated translational control	Deletion	Reduced cyst formation	[43]
Alba2	Alba1-dependent target	Deletion	No effect	[43]
PUS1	RNA pseudouridylation	Deletion	Defective differentiation; smaller cysts	[47,108]
Cdc5	Core splicing factor	Conditional knockdown (mAID)	Abortive differentiation	[111]
SR2	Serine-arginine-rich splicing factor	Deletion	↓80% cysts *in vivo*	[113]
SR3	Serine-arginine-rich splicing factor	Overexpression	Differentiation role unclear	[112]
ncRNA-1	Developmentally regulated lncRNA	Insertional disruption	Blocks differentiation	[117]
METTL3	m6A methyltransferase	Depletion	Essential for viability; differentiation role unclear	[128,129]
CPSF4	m6A reader	Depletion	Essential for viability; differentiation role unclear	[129]

### Translation initiation

#### Phosphorylation of eIF2α promotes bradyzoite formation

One of the best-characterized mechanisms of translational control during *T. gondii* differentiation is the phosphorylation of the eukaryotic initiation factor 2 alpha subunit (eIF2α), a hallmark of the integrated stress response (ISR) [91]. In eukaryotic cells, eIF2α phosphorylation inhibits eIF2B, a guanine nucleotide exchange factor required to regenerate eIF2-GTP from eIF2-GDP [92]. This limits ternary complex formation, broadly suppressing global protein synthesis while allowing preferential translation of stress-responsive mRNAs that promote cellular adaptation and survival.

In *T. gondii*, eIF2α is phosphorylated in response to multiple stressors, including ER stress and oxidative stress, and this modification promotes the developmental switch from the rapidly replicating tachyzoite to the latent bradyzoite stage [93] (Figure 4). Importantly, eIF2α remains phosphorylated in mature bradyzoites [42], suggesting a long-term repression of bulk translation is necessary to maintain latency.

*T. gondii* encodes multiple stress-responsive kinases that phosphorylate eIF2α under distinct conditions [42,94,95]. eIF2K-A, a PERK-like kinase localized to the endoplasmic reticulum (ER), is activated under ER stress conditions such as exposure to tunicamycin and calcium ionophore [42]. Its activation mimics features of mammalian PERK, including dissociation from the ER chaperone BiP, and triggers global translational repression while promoting bradyzoite gene expression [42]. Consistent with this role, treatment with the PERK-specific inhibitor GSK2606414 blocks eIF2K-A kinase activity, reduces eIF2α phosphorylation under ER stress, and significantly reduces the frequency of bradyzoite differentiation *in vitro* [96].

eIF2K-B, in contrast, is activated by oxidative stress (e.g., sodium arsenite). Deletion of *eif2k-b* results in elevated reactive oxygen species, enhanced tachyzoite replication, and severely impaired bradyzoite differentiation [94]. Transcriptomic analysis of Δ*eif2k-b* parasites reveals downregulation of differentiation regulators (e.g., *BFD1*) and upregulation of replication-associated factors (e.g., *AP2IX-9*), shifting parasites toward a proliferative state [94]. These parasites are more virulent during acute infection but show reduced cyst burden during chronic infection, highlighting the role of redox signaling in stage conversion [94].

Two additional GCN2-like kinases, eIF2K-C and eIF2K-D, respond to nutrient deprivation, with eIF2K-C functioning during intracellular amino acid limitation and eIF2K-D supporting extracellular survival following egress [95]. While their roles in bradyzoite development remain unclear, they highlight the broader importance of translational control in stress adaptation [95].

Pharmacologic studies further support the importance of sustained eIF2α phosphorylation in *T. gondii* differentiation. Small-molecule inhibitors of eIF2α dephosphorylation, such as salubrinal and guanabenz, induce bradyzoite formation even in the absence of stress [93]. *In vitro*, these compounds suppress tachyzoite replication, promote latency, and block bradyzoite reactivation upon stress removal [93]. *In vivo*, guanabenz extends survival, reduces brain cyst burden, and mitigates behavioral changes in infected mice [97,98]. Interestingly, bradyzoites formed under prolonged treatment with these compounds often display abnormal morphology, suggesting that precise control of translational repression is essential for cyst integrity [97].

Together, these findings underscore the importance of tightly regulated eIF2α phosphorylation in both stage conversion and maintenance of latency and highlight translational control as a promising therapeutic target for toxoplasmosis.

#### eIF1.2 is essential for bradyzoite formation

Beyond global translational repression mediated by eIF2α phosphorylation, recent studies have revealed that additional components of the translation initiation machinery, such as eIF1.2, play a critical and selective role in stage-specific gene expression during *T. gondii* differentiation (Figure 4). Identified through a forward genetic screen, eIF1.2 is a paralog of canonical eIF1 and is conserved among tissue cyst-forming apicomplexans [45].

A single point mutation (F97L) in eIF1.2 does not impair type II tachyzoite growth *in vitro* or during acute infection in mice. In contrast, complete deletion of eIF1.2 in type II parasites significantly impairs tachyzoite growth *in vitro*. Importantly, both the F97L mutation and genetic deletion of *eif1.2* markedly impaired bradyzoite cyst formation *in vitro* and *in vivo*, underscoring the essential role of eIF1.2 in establishing chronic infection [45,99].

Mechanistically, eIF1.2 promotes translation initiation fidelity during ribosomal scanning. Single-molecule fluorescence assays show that the F97L mutation alters 48S complex scanning by increasing bypass of near-cognate start codons, impairing accurate start codon recognition [45]. Ribosome profiling and transcriptomic analyses demonstrate that Δ*eif1.2* parasites fail to upregulate key differentiation factors under stress conditions. Specifically, under alkaline stress, loss of eIF1.2 impairs the stress-induced translational upregulation of the master regulator BFD1, as well as the transcriptional and translational upregulation of its cofactor BFD2 [45]. Conditional stabilization of BFD1 or BFD2 partially restores cyst formation in ∆*eif1.2* parasites, positioning these factors downstream of eIF1.2 in the differentiation pathway [45]. Together, these findings indicate that eIF1.2 functions as a gatekeeper for the selective translation of differentiation-promoting transcripts in response to stress.

Intriguingly, *T. gondii* encodes a second eIF1 paralog, eIF1.1, which shares approximately 82% sequence identity with eIF1.2. However, deletion of *eif1.1* in the RH strain does not affect tachyzoite growth, invasion, replication, egress, or the differentiation response to alkaline stress, suggesting that eIF1.1 is dispensable for both the lytic cycle progression and stage conversion [99]. eIF1.1 expression is dynamically regulated during differentiation. In wild-type parasites, eIF1.1 is downregulated under conditions that trigger bradyzoite formation. However, in ∆*eif1.2* parasites, eIF1.1 levels remain unchanged under the same conditions, suggesting that eIF1.1 is not sufficient to compensate for the loss of eIF1.2 during differentiation [45]. This functional divergence underscores eIF1.2’s specialized role in coupling translation initiation fidelity to stage conversion in *T. gondii*, while raising new questions about the potential distinct functions of eIF1.1.

#### Repression of eIF4E1 triggers bradyzoite formation

Recent studies have also identified eIF4E1, the primary mRNA cap-binding protein of the eIF4F complex, as a critical regulator of *T. gondii* stage conversion [44] (Figure 4). The eIF4F complex mediates cap-dependent translation initiation by recruiting the 43S complex to the 5′ end of mRNAs to form the 48S complex [52,100–102]. Biochemical and transcriptomic evidence confirms that eIF4E1 is the dominant cap-binding protein in tachyzoites. Crosslinking immunoprecipitation (CLIP-seq) and m^7^G-cap affinity purification assays demonstrate that *T. gondii* eIF4E1 associates with a broad subset of protein-coding transcripts at their 5' ends [44]. These binding sites often coincide with transcriptional start sites (TSSs) and reveal multiple peaks indicative of transcriptional heterogeneity, which may regulate translation through alternative 5′-leaders [44].

Like mammalian eIF4E1, *T. gondii* eIF4E1 assembles into functional eIF4F complexes containing eIF4G1/2, eIF4A, and PABP [44]. Furthermore, like its mammalian counterparts, *T. gondii* eIF4E1 associates with actively translating ribosomes under unstressed conditions and redistributes to stress granules during oxidative stress, underscoring its conserved yet dynamic role in translation regulation [44]. Genetic depletion of eIF4E1 using an auxin-inducible degron (AID) system significantly reduces global protein synthesis and parasite replication [44]. Ribosome profiling data [44] show preferential repression of ribosomal protein transcripts and other translation-related mRNAs, especially those with shorter 5′-UTRs, suggesting potential parallels to mammalian mTOR-regulated translation, despite the absence of canonical 5′-TOP motifs in *T. gondii*.

Remarkably, loss of eIF4E1 in both type I (RH) and type II (ME49) strains is sufficient to induce spontaneous bradyzoite formation, even in the absence of external stress [44]. In RH parasites, which typically resist stress-induced encystation, eIF4E1 depletion was more effective than alkaline stress. Furthermore, chemical inhibition of eIF4E1–eIF4G interactions using the small molecule 4EGI-1 similarly induced bradyzoite differentiation in both RH and ME49 strains, demonstrating that disruption of eIF4F complex assembly is a potent trigger for *T. gondii* stage conversion [44].

Transcriptomic profiling confirmed that eIF4E1 depletion leads to a shift toward bradyzoite-like gene expression, mirroring profiles seen under alkaline stress [44]. Interestingly, while RH parasites were able to recover from bradyzoite formation following transient eIF4E1 depletion, ME49 parasites were not, suggesting strain-specific differences in eIF4E1-driven plasticity during differentiation [44].

These results position eIF4E1 as a regulator of the tachyzoite-to-bradyzoite switch, acting through selective translational control of growth- and stage-specific genes. Together with findings on eIF2α and eIF1.2, this work reveals a multi-tiered network of translation initiation factors that coordinate *T. gondii* differentiation and latency [44,45,93].

### RNA-binding proteins

#### BFD2/ROCY1 licenses commitment to differentiation through translational activation of BFD1

**Figure 5:**
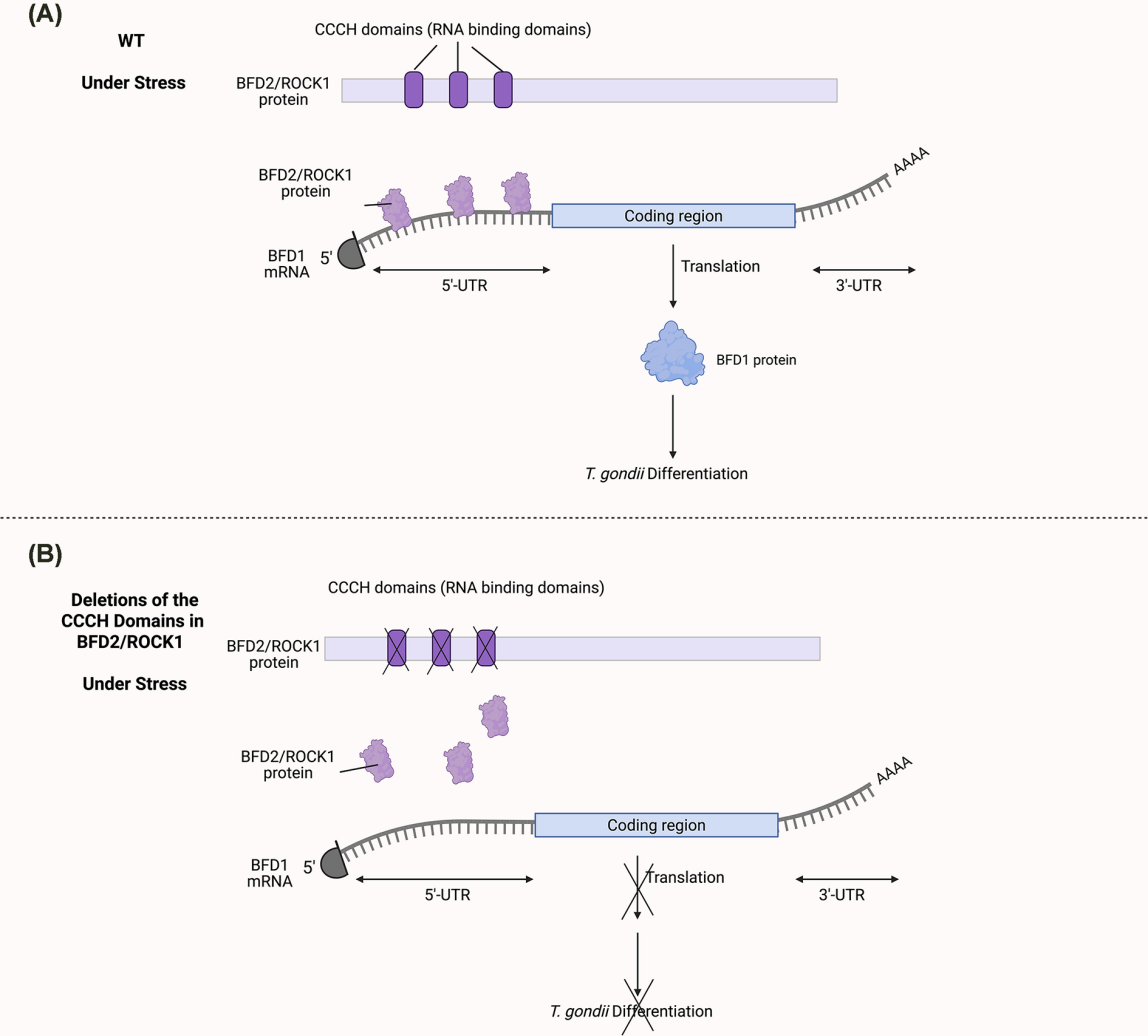
BFD2/ROCY1-mediated translational activation of BFD1 reinforces commitment to *T. gondii* differentiation (**A**) Under differentiation-inducing stress, the CCCH-type RBP BFD2/ROCY1 binds the BFD1 transcript, as demonstrated by RNA immunoprecipitation and eCLIP-based analyses, thereby promoting BFD1 protein translation without altering mRNA abundance. This translational activation initiates the BFD1-dependent transcriptional program driving bradyzoite differentiation and cyst formation [46,103,104]. (**B**) Genetic disruption of BFD2/ROCY1 or deletion of its RNA-binding domains markedly reduces BFD1 protein levels and impairs differentiation, whereas BFD2/ROCY1 overexpression is sufficient to induce differentiation in the absence of stress. These findings support a positive feedback loop in which BFD1 transcriptionally upregulates BFD2/ROCY1, which in turn enhances BFD1 translation [46,103,104].

In addition to canonical translation initiation factors, *T. gondii* utilizes specialized RBPs to tightly regulate stage-specific protein synthesis. One such protein, Bradyzoite Formation Deficient 2 (BFD2), also known as Regulator of Cystogenesis 1 (ROCY1), is a cytosolic RBP containing CCCH-type zinc finger domains [46,103]. BFD2/ROCY1 is both necessary and sufficient to drive *T. gondii* differentiation into the chronic stage [46,103] (Figure 5).

During stress-induced differentiation, BFD2/ROCY1 binds to numerous transcripts, including *BFD1*, the master transcription factor required for chronic stage formation [46,103]. Although BFD1 mRNA is constitutively transcribed, its translation is repressed in tachyzoites. Under differentiation-inducing conditions such as alkaline stress, BFD2/ROCY1 binds to regions within the 5′-UTR and coding sequence of *BFD1* mRNA, promoting its translation without altering transcript abundance [46,103] (Figure 5A). Loss of BFD2/ROCY1 prevents BFD1 protein production, thereby blocking downstream transcriptional reprogramming and halting *T. gondii* differentiation despite continued *BFD1* mRNA expression [46]. RNA immunoprecipitation experiments demonstrate that this regulatory interaction depends on the integrity of the CCCH zinc finger domains of BFD2/ROCY1 [46,103]. Deletion of these domains disrupts the binding of BFD2/ROCY1 to *BFD1* mRNA, preventing BFD1 protein accumulation and thereby blocking differentiation even under inducing conditions [46] (Figure 5B).

Strikingly, overexpression of BFD2/ROCY1 alone is sufficient to induce differentiation in the absence of stress, underscoring its instructive role in developmental fate decisions [46,103]. Conversely, site-directed mutagenesis of the zinc finger domains abolishes this effect, confirming that RNA binding is essential for BFD2/ROCY1 activity [103].

Importantly, BFD1 transcriptionally upregulates *BFD2*/*ROCY1*, establishing a positive feedback loop [104]. This circuit reinforces the chronic differentiation program: basal BFD2 levels enable BFD1 translation, and BFD1, in turn, amplifies BFD2 expression, locking the system into a committed bradyzoite state. Disruption of either BFD1 or BFD2 impairs this self-reinforcing loop and abolishes robust cyst formation [46,104].

Although BFD2 promotes differentiation and cyst formation, its complete loss does not completely eliminate parasite persistence *in vivo*. Parasites lacking BFD2 or BFD1 form significantly fewer cysts and show diminished bradyzoite gene expression, yet they can still persist in the brains of infected mice and cause recrudescent disease upon immunosuppression [46,103]. These findings challenge the long-held assumption that canonical cyst formation is strictly required for long-term persistence and reactivation.

Together, these studies identify BFD2/ROCY1 as a key translational activator that regulates chronic stage formation through translational activation of BFD1. The BFD1–BFD2 feedback loop likely contributes to bistability and hysteresis in the differentiation circuit, ensuring that once the switch to bradyzoite formation is activated, the commitment is maintained even after stress subsides. Future studies are needed to determine how this regulatory circuit is disengaged during reactivation and whether BFD2 also regulates additional transcripts involved in non-cyst persistence.

#### Alba: a translational regulator required for stress-induced differentiation

Alba1 and Alba2 are conserved RBPs of the ALBA (Acetylation Lowers Binding Affinity) family, known for roles in RNA metabolism, stress responses, and developmental regulation across diverse organisms [105]. In *T. gondii*, these proteins participate in a translation-associated network that responds to environmental stress [43].

Under normal conditions, both Alba1 and Alba2 localize to the cytoplasm of intracellular parasites. Upon extracellular stress, they relocalize to perinuclear RNA granules, suggesting a role in post-transcriptional regulation during adaptation [43].

**Figure 6:**
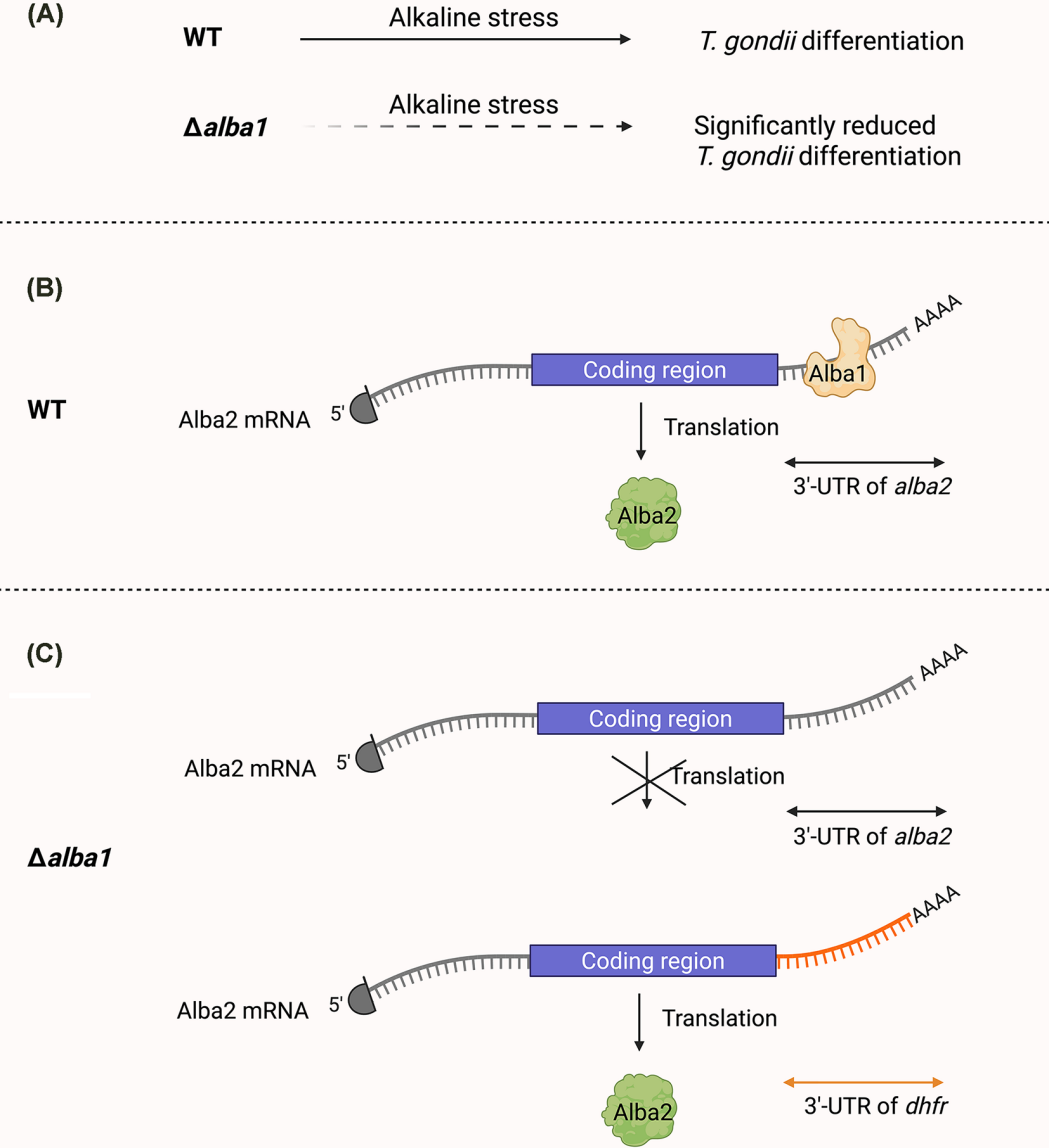
Alba1 regulates *T. gondii* differentiation and Alba2 translation via 3'-UTR-dependent post-transcriptional control (**A**) Targeted genetic deletion of *Alba1* impairs alkaline stress-induced bradyzoite differentiation *in vitro* and reduces cyst formation *in vivo*, as quantified by cyst wall lectin staining, parasite growth assays, and mouse infection models [43]. (**B**) Tandem affinity proteomic purification identified Alba1 and Alba2 within complexes containing translation machinery components, including cap-binding proteins and eIF3 subunits, supporting a role in translational regulation. RNA immunoprecipitation coupled with microarray profiling demonstrated association of Alba proteins with a defined subset of mRNAs, including *Alba1* and *Alba2* transcripts [43]. (**C**) Despite elevated *Alba2* mRNA levels, loss of Alba1 abolishes detectable Alba2 protein expression, as shown by immunoblotting and fluorescence microscopy, establishing post-transcriptional control. 3'-UTR-replacement knock-in experiments further revealed that Alba2 translation requires both the native 3′-UTR and Alba1, identifying a 3′-UTR-dependent mechanism for gene-specific translational regulation during stress-induced differentiation [43].

Functional studies show that Alba1 is essential for *T. gondii* differentiation under alkaline stress. Δ*alba1* parasites exhibit reduced cyst wall formation *in vitro* and lower cyst burden *in vivo*, indicating a failure to respond to stress cues (Figure 6A). In contrast, Alba2 is dispensable for differentiation, despite its expression depending on Alba1.

Specifically, Alba1 is required for translation, but not transcription, of *alba2* (Figure 6B). In Δ*alba1* parasites, *alba2* mRNA levels are elevated, but the Alba2 protein is undetectable. This translational repression is dependent on the native 3′-UTR of *alba2* mRNA: replacing it with the 3′-UTR of *dhfr* restores Alba2 protein synthesis even in the absence of Alba1 [43] (Figure 6C). These findings reveal a 3′-UTR-dependent mechanism through which Alba1 facilitates translation of target mRNAs.

Tandem affinity purification of Alba1 and Alba2 identified shared interactions with components of the translation machinery, including ribosomal subunits, poly(A)-binding proteins, eIF3 subunits, and cap-binding factors. RNA pull-down and microarray analyses revealed that both proteins associate with approximately 30 mRNAs [43], supporting a model of transcript-specific translational regulation.

Alba1 may enhance translation through mechanisms such as promoting ribosome recruitment, stabilizing mRNA structure, or alleviating 3'-UTR-mediated repression. Although Alba2 is one of Alba1’s regulatory targets, it is not required for stage conversion. Instead, Alba1 likely governs translation of a broader set of stress-responsive genes critical for differentiation.

In summary, Alba1 is a key translational regulator that promotes bradyzoite differentiation through 3′-UTR-mediated control of gene expression. Its role underscores the importance of selective translation in parasite adaptation and highlights Alba1 as a potential target for disrupting chronic *T. gondii* infection.

### Pseudouridylation as a post-transcriptional switch for *T. gondii* differentiation

RNA modifications are increasingly recognized as important regulators of gene expression in eukaryotes [106]. Among these, pseudouridylation (Ψ), the isomerization of uridine to pseudouridine, is one of the most abundant and conserved modifications, known to influence RNA structure, stability, and function [107]. In *T. gondii*, pseudouridylation has been implicated as an important regulator of developmental transitions between the tachyzoite and bradyzoite stages [47].

**Figure 7:**
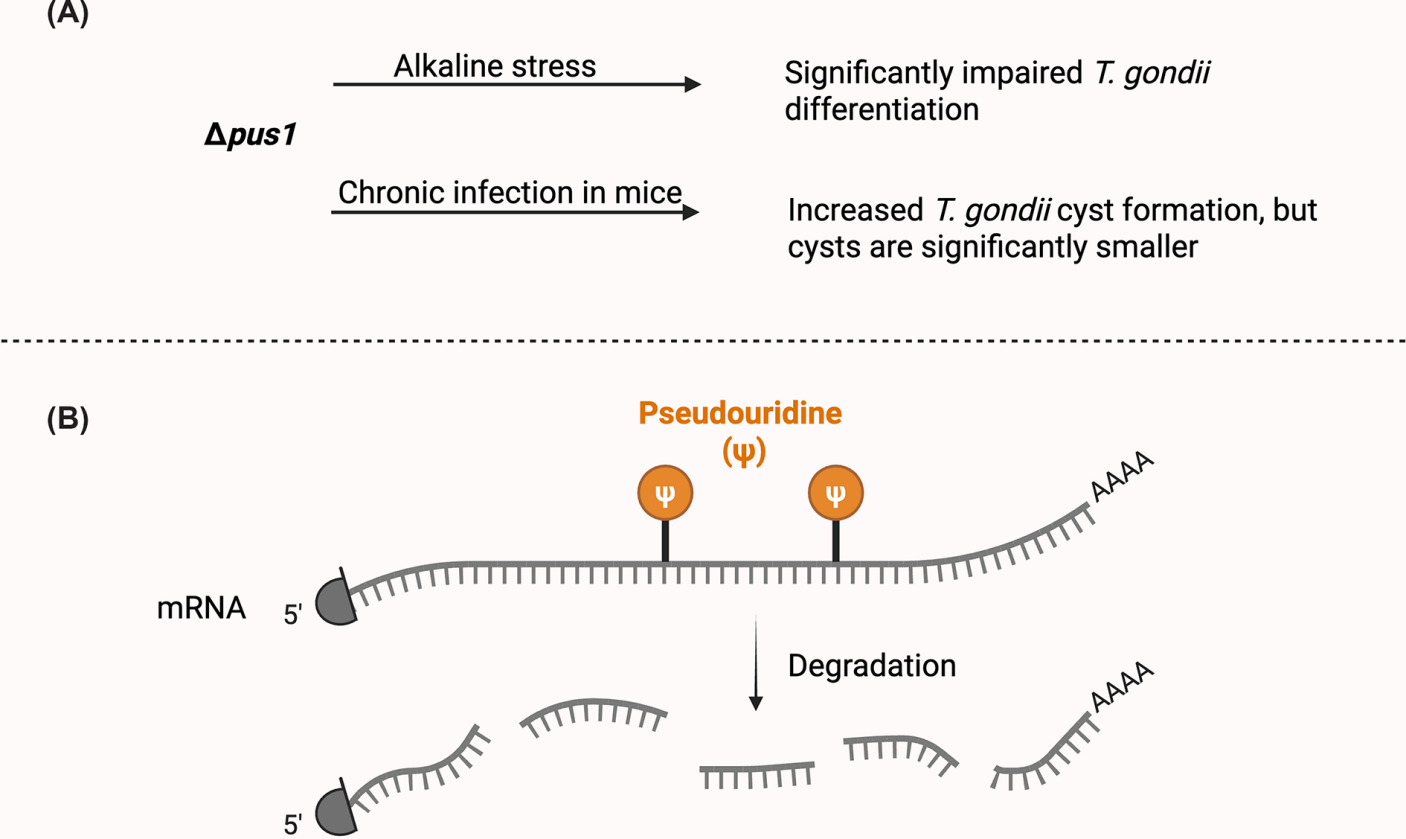
PUS1-mediated pseudouridylation regulates *T. gondii* differentiation and mRNA stability (**A**) Genetic disruption of PUS1 (insertional mutants and targeted knockout) impairs bradyzoite differentiation *in vitro* under alkaline stress, as assessed by cyst wall lectin staining, and alters developmental outcomes in vivo, resulting in increased cyst numbers but reduced cyst size [47]. (**B**) Transcriptome-wide pseudouridine mapping using CMCT-based PSI-seq identified PUS1-dependent Ψ modifications across mRNAs and tRNAs. RNA decay measurements using 4-thiouracil pulse-chase labeling showed that loss of PUS1 modestly increased the stability of a subset of transcripts harboring PUS1-dependent pseudouridylation, indicating that PUS1-mediated RNA modification contributes to transcript-specific regulation of mRNA stability during stage conversion [108].

The pseudouridine synthase PUS1 is indispensable for efficient differentiation. Parasites lacking PUS1 replicate more slowly and show impaired bradyzoite formation under stress conditions *in vitro*. *In vivo*, these mutants display increased acute virulence and generate a higher number of smaller cysts in chronically infected mice [47] (Figure 7A), underscoring the importance of RNA modification in regulating developmental plasticity.

Transcriptome-wide pseudouridine mapping using pseudouridine site identification sequencing (PSI-seq) revealed over 1600 Ψ sites in tachyzoite mRNAs and several hundred in bradyzoites. These modifications are non-randomly distributed across the 5′-UTR, coding region, and 3′-UTR, though they are significantly depleted in the 3′-UTR compared with uridines [108]. Within coding region, Ψ is enriched at the first codon position while being less frequent at positions 2 and 3 [108]. The distinct distribution suggests potential roles in translational regulation and mRNA stability. Notably, a subset of Ψ sites is PUS1-dependent and enriched in mRNAs highly expressed during the tachyzoite stage [108]. In addition to mRNAs, PUS1 also catalyzes pseudouridylation of specific residues in tRNAs, particularly in the anticodon stem-loop [108], further supporting its broad role in shaping the parasite’s translational landscape.

Unlike other pseudouridine synthases that recognize specific sequence motifs, PUS1 appears to act without a strong consensus motif [108]. Loss of PUS1-dependent pseudouridines leads to a modest but statistically significant increase in mRNA abundance, particularly for transcripts harboring Ψs in 5′-UTRs or coding regions [108]. Pulse-chase labeling with 4-thiouracil (4TU) further showed that PUS1-mediated pseudouridylation modestly reduces mRNA stability (Figure 7B), suggesting a role in regulating transcript turnover [108].

Together, these findings establish pseudouridylation as an epitranscriptomic mechanism contributing to post-transcriptional and translational regulation in *T. gondii* [108]. Through fine-tuning of mRNA and tRNA function, PUS1-mediated pseudouridylation orchestrates stage-specific gene expression and contributes to the parasite’s developmental flexibility [47,108]. These studies significantly advance our understanding of how RNA modifications interface with transcriptional and translational networks to coordinate developmental transitions in apicomplexan parasites.

### Splicing factors and alternative splicing in *T. gondii* differentiation

**Figure 8:**
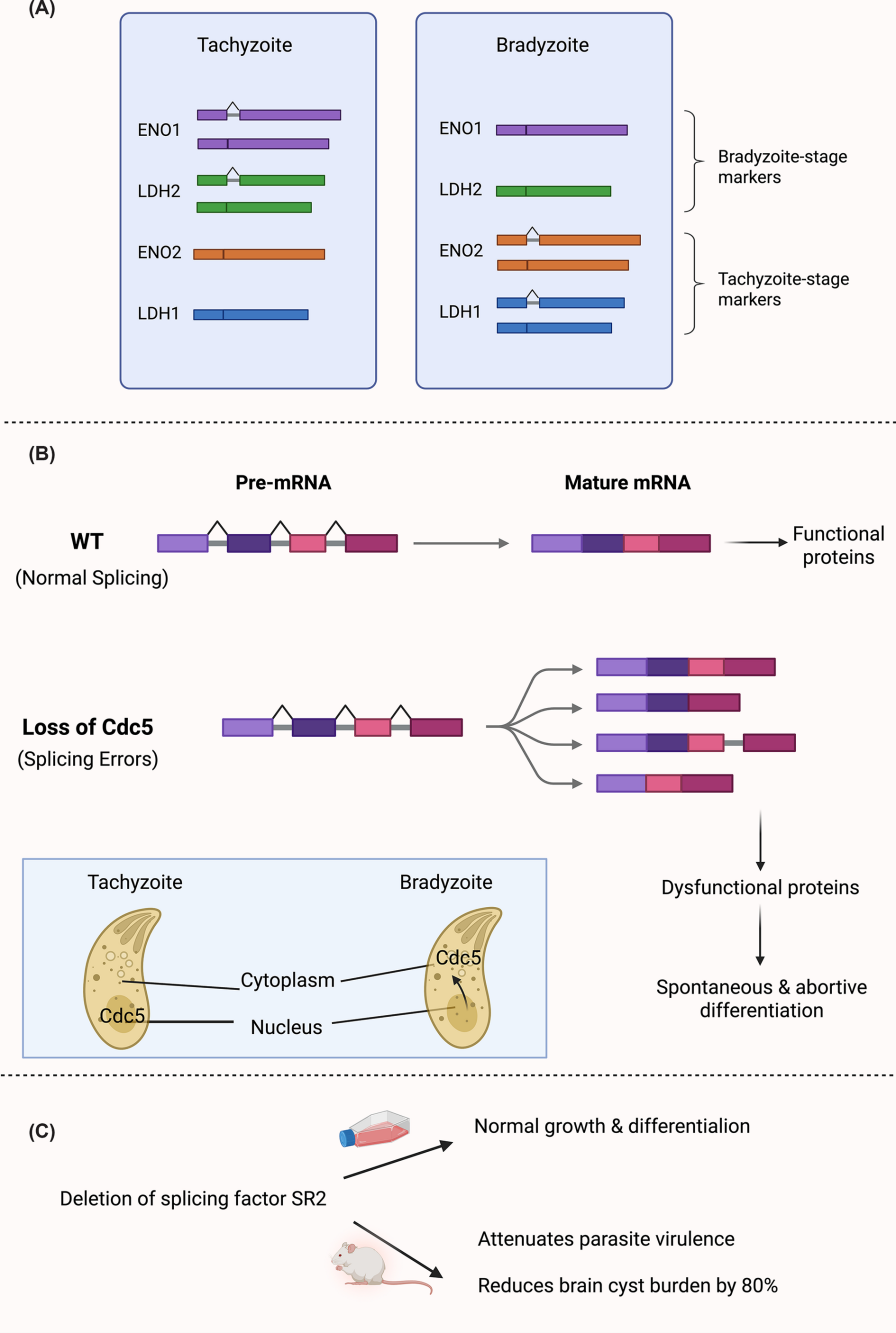
Splicing-dependent post-transcriptional regulation in *T. gondii* differentiation and pathogenicity (**A**) Stage-specific intron retention in glycolytic transcripts encoding ENO1/ENO2 and LDH1/LDH2 was demonstrated by RT-PCR and transcript analyses, revealing that inappropriate isoforms are expressed as unspliced, premature stop codon-containing transcripts during stage transition. These transcripts are not expressed as proteins at the non-permissive stage, suggesting that intron retention helps prevent untimely enzyme expression during differentiation [110]. (**B**) Loss of the spliceosomal factor Cdc5 causes global splicing failure dominated by intron retention (as shown by RNA-seq and reporter assays), leading to dysfunctional gene expression programs that induce spontaneous but abortive bradyzoite differentiation and loss of parasite viability [111]. (**C**) CRISPR–Cas9 deletion of the SR splicing factor SR2 reveals no defects in parasite growth or *in vitro* bradyzoite differentiation, as assessed by plaque, replication, egress, and cyst formation assays, but causes strong attenuation of virulence and approximately 80% reduction in brain cyst burden in mouse infection models, demonstrating a key role for SR2-mediated splicing in *T. gondii* pathogenicity [113].

Alternative splicing expands proteomic diversity and fine-tunes gene expression during cellular adaptation and developmental transitions. In *T. gondii*, over 75% of genes contain introns, in contrast to only 5% in yeast, suggesting that splicing regulation may play an important role in its complex life cycle [109]. This regulation is achieved through multiple layers of control, including stage-specific intron retention (IR) [110]. IR functions as a molecular switch, particularly for key metabolic enzymes. For example, transcripts of glycolytic enzymes *ENO1*, *ENO2*, *LDH1*, and *LDH2* exhibit stage-specific splicing: the bradyzoite-specific isoforms (ENO1 and LDH2) retain introns in tachyzoites, while the tachyzoite-specific isoforms (ENO2 and LDH1) retain introns in bradyzoites. These unspliced transcripts are largely translationally inactive, preventing inappropriate protein expression [110] (Figure 8A). Furthermore, for ENO2, the presence and proper splicing of the intron are required to achieve normal mRNA abundance, establishing splicing as a prerequisite for translation.

The critical importance of splicing machinery is underscored by the recent identification of the conserved splicing factor Cdc5 in* T. gondii* [111]. Cdc5 is part of a large spliceosomal complex containing at least 52 putative proteins. It localizes to the nucleus in tachyzoites but relocalizes to the cytoplasm during the bradyzoite stage (Figure 8B), and functionally complements yeast lacking its ortholog, demonstrating evolutionary conservation [111].

Depletion of Cdc5 causes severe, genome-wide splicing defects, with intron retention accounting for over 82% of all splicing events and affecting nearly 65% of genes [111]. This widespread disruption leads to profound phenotypic consequences, including impaired parasite replication, invasion, and egress; defective DNA replication and repair; loss of protein homeostasis; cell cycle arrest; and increased apoptosis-like death [111].

Strikingly, Cdc5 depletion in the non-cystogenic RH strain triggers spontaneous bradyzoite formation without external stress, characterized by upregulation of bradyzoite activators (AP2IV-3, AP2IX-9, AP2XI-4) and markers (BAG1, CST1, IF2K-A, HSP60, MAG1), alongside the downregulation of bradyzoite repressors (AP2IV-4, AP2XII-2) [111]. However, these bradyzoites are abortive: they fail to form mature cysts, exhibit disintegrated cyst walls, and cannot revert to tachyzoites [111] (Figure 8B). This phenotype likely reflects widespread intron retention, which prevents the production of functional proteins required for bradyzoite maintenance.

In addition to core factors such as Cdc5, *T. gondii* encodes at least four serine/arginine-rich (SR) splicing factors (SR1–SR4), with SR3 modulating alternative splicing in more than 1,000 genes [112]. Although deletion of SR2, a homolog of *Plasmodium* SR1, does not affect tachyzoite growth or bradyzoite differentiation *in vitro*, it significantly attenuates parasite virulence and reduces brain cyst burden by approximately 80% *in vivo* [113]. This finding suggests that splicing regulation by SR2 may integrate host-specific cues or immune pressures absent in standard cell culture models [113], further highlighting the importance of precise splicing control for successful infection.

The essentiality of the splicing machinery is underscored by its potential as a therapeutic and vaccine target. Cdc5 is essential for parasite survival *in vivo*. Mice infected with Cdc5-mAID-HA parasites and treated with IAA showed complete protection from lethal toxoplasmosis, with no detectable parasites in brain or heart tissues [111]. Moreover, exposure to these replication-defective parasites generated a robust immune response that conferred complete protection against future acute infection and partial protection during pregnancy [111].

Together, core spliceosomal factors (Cdc5), SR proteins, and stage-specific intron retention enable *T. gondii* to coordinate mRNA maturation and translation with developmental cues. The essentiality of splicing factors, the abortive bradyzoite phenotype observed upon their disruption, and the protective immune response elicited by splicing-deficient parasites position the spliceosome as a promising therapeutic target and highlights the potential of splicing-deficient attenuated strains for vaccine development.

### Non-coding RNAs as regulators of translational control in *T. gondii* differentiation

A substantial portion of the *T. gondii* genome is transcribed into non-coding RNAs (ncRNAs), including long non-coding RNAs (lncRNAs) and diverse small RNAs such as microRNAs (miRNAs) and small interfering RNAs (siRNAs) [114]. Growing evidence indicates that these ncRNAs contribute to parasite differentiation by shaping post-transcriptional gene regulation, particularly through control of mRNA stability, ribonucleoprotein complex assembly, and access of transcripts to the translation machinery [114].

*T. gondii* retains the core components of an RNA silencing machinery, including Dicer, Argonaute (AGO), and a putative RNA-dependent RNA polymerase [115], which together mediate RNA interference (RNAi), a process in which small RNAs guide the degradation or translational repression of target mRNAs. Deep sequencing revealed a diverse small RNA population composed of metazoan-like miRNAs and repeat- and satellite-derived siRNAs, both of which are loaded onto AGO. Repeat-associated small RNAs map to heterochromatic regions enriched in silencing markers, suggesting roles in genome surveillance, while several miRNAs associate with polysomes and are proposed to regulate gene expression at the translational level [115]. However, despite the presence of RNAi components, double-stranded RNA-mediated gene silencing has proven difficult to reproduce consistently across laboratories [116].

**Figure 9:**
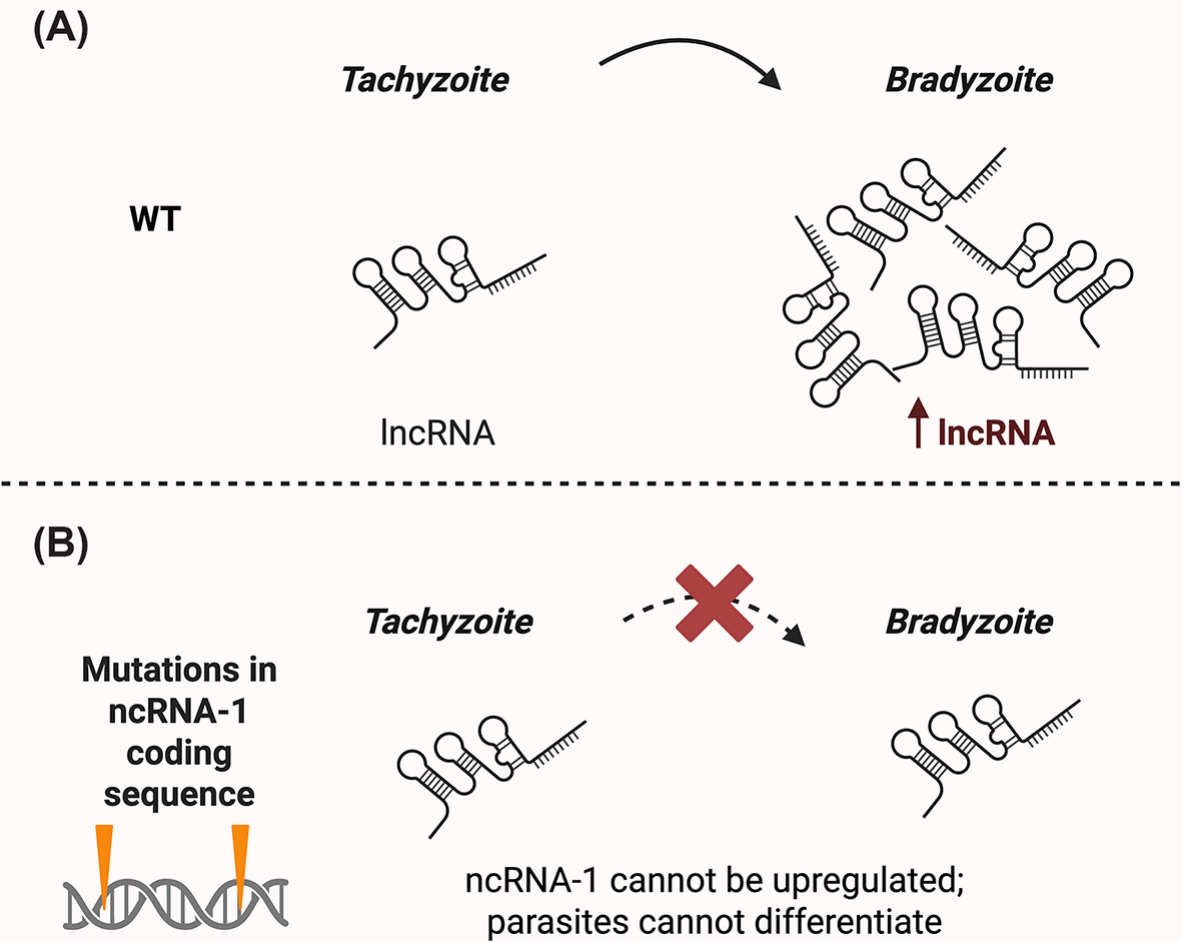
Developmental regulation of ncRNA-1 is required for bradyzoite formation (**A**) In wild-type parasites, ncRNA-1 is a developmentally regulated transcript that is upregulated during tachyzoite-to-bradyzoite differentiation, coinciding with successful stage conversion, as demonstrated by qRT-PCR analysis [117]. (**B**) In parasites carrying insertional disruptions within the ncRNA-1 locus, ncRNA-1 expression is reduced and parasites fail to efficiently undergo differentiation. This defect is evidenced by reduced expression of bradyzoite markers (e.g., BAG1 and cyst wall lectin staining), continued parasite replication under differentiation conditions, and impaired induction of bradyzoite gene expression programs. Complementation with the wild-type ncRNA-1 locus restores normal differentiation, demonstrating its functional requirement [117].

The strongest functional link between ncRNAs and differentiation is provided by ncRNA-1, a developmentally regulated lncRNA identified in insertional mutagenesis screens for bradyzoite-defective mutants (Figure 9). ncRNA-1 expression is strongly induced during tachyzoite-to-bradyzoite conversion (∼24-fold), and its disruption severely impairs bradyzoite formation, prevents growth arrest under differentiation conditions, and blocks induction of the majority of stage-specific genes [117]. Complementation restores differentiation, confirming locus-specific function [117]. Notably, ncRNA-1 contains sequences corresponding to repeat-derived small RNAs that associate with AGO in *T. gondii* [115], suggesting that it may act as a precursor for regulatory small RNAs involved in post-transcriptional or chromatin-based repression during stage conversion. Alternatively, ncRNA-1 may function as a long ncRNA scaffold recruiting epigenetic regulators to developmental loci [117].

More broadly, a recent bioinformatic analysis predicted 656 putative lncRNAs in *T. gondii*, with 217 differentially expressed during stage conversion, including 214 strongly enriched in the bradyzoite stage [118]. Co-expression clustering placed subsets of these lncRNAs alongside canonical bradyzoite markers (*BAG1*, *ENO1*, *LDH2*) and the differentiation-associated RBP BFD2, suggesting association with developmental expression programs [118]. Genomic mapping revealed that most lncRNA loci lie within 10 kb of protein-coding gene transcription start sites, supporting widespread *cis*-regulatory potential. In parallel, predicted RNA–RNA interaction networks linked distally located lncRNAs with transcripts encoding Argonaute, Dicer, ApiAP2 transcription factors, and DNA methyltransferase, consistent with possible *trans*-acting regulatory roles [118]. Comparative motif analysis identified NORAD-like features of a stress-responsive lncRNA that modulates translation by sequestering Pumilio RBPs, together with conserved Pumilio-binding elements in several *T. gondii* lncRNAs, implicating lncRNA–RBP networks in stress-responsive translational control during differentiation [118,119].

Collectively, ncRNAs provide sequence-specific and dynamic regulation of translational output during *T. gondii* differentiation. Elucidating how these ncRNA networks interface with RBPs and ribosomal control pathways remains essential for defining the molecular basis of developmental proteome reprogramming.

## Unexplored mechanisms of translational control in *T. gondii* differentiation

**Figure 10:**
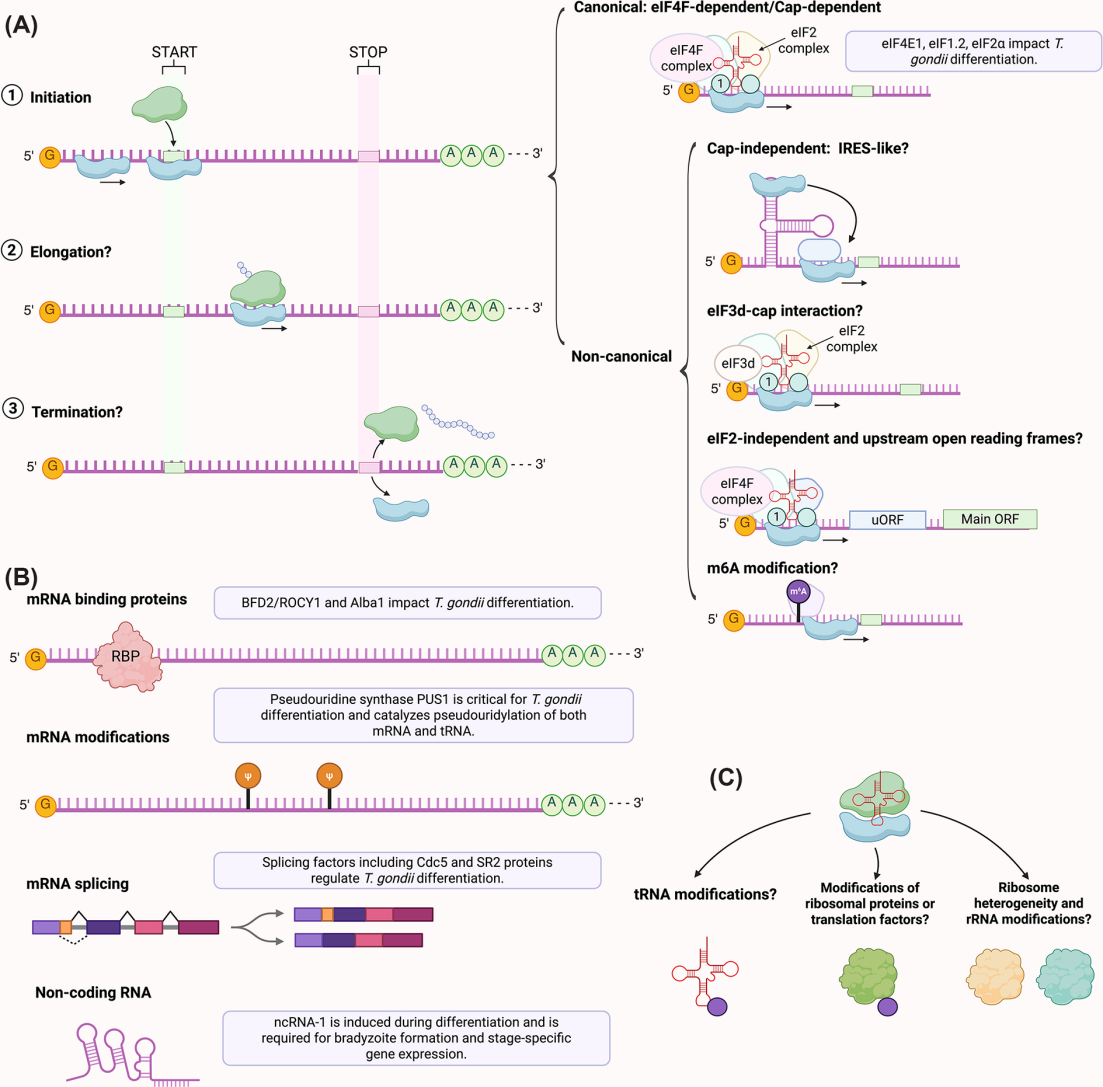
Established and emerging mechanisms of translational regulation in *T. gondii* differentiation (**A**) Translation proceeds through initiation, elongation, and termination. Canonical cap-dependent initiation is an established regulator of stage conversion and involves eIF4F-mediated cap recognition and recruitment of the eIF2 ternary complex to form the 43S pre-initiation complex. Genetic and pharmacologic studies demonstrate that eIF4E1, eIF1.2, and stress-induced phosphorylation of eIF2α regulate bradyzoite differentiation [42,44,45,93]. In contrast, several non-canonical initiation mechanisms remain largely untested in *T. gondii*, including IRES-like cap-independent initiation, eIF3d-dependent cap binding, eIF2-independent initiation, uORF-mediated translational tuning, and m6A-mediated ribosome recruitment. (**B**) Post-transcriptional mechanisms with direct experimental support include pseudouridylation by PUS1 (demonstrated by genetic deletion and RNA modification mapping) [47,108], RNA-binding protein-mediated translational control by BFD2/ROCY1 and Alba1 (defined by RIP-seq, reporter assays, and knockout phenotypes) [43,46,103], and stage-specific mRNA splicing that governs translational competence [110,111]. (**C**) Additional regulatory layers, such as tRNA modifications, PTM of translation factors, ribosome heterogeneity, and rRNA modifications, are proposed based on studies in other systems but remain largely unexplored in *T. gondii* differentiation.

While recent work has established translation initiation factors, RNA-binding proteins, RNA modifications, alternative splicing, and ncRNAs as regulators of stage conversion, additional layers of translational control remain largely unexplored in *T. gondii*. Studies in diverse organisms demonstrate that developmental transitions are often accompanied by extensive remodeling of translation initiation strategies, ribosome composition, and post-initiation control steps. Whether similar mechanisms operate during *T. gondii* differentiation represents a key frontier (Figure 10).

### Cap-independent and alternative translation initiation pathways

Although cap-dependent translation mediated by eIF4E1 is critical for *T. gondii* differentiation, stress conditions probably engage alternative initiation mechanisms to sustain selective protein synthesis. In other eukaryotes, internal ribosome entry sites (IRES) support translation when cap-dependent initiation is compromised, but functional IRES elements have not yet been identified in *T. gondii.*

BFD1 translation can be maintained when cap-dependent translation is reduced, suggesting that noncanonical initiation pathways may already operate in the parasites [120]. One such mechanism involves eIF3d-mediated cap recognition, which bypasses eIF4E under stress [121]. A putative eIF3d homolog (TGME49_317720) is present in the *T. gondii* genome, but its function remains uncharacterized. In addition, m^6^A modifications within 5′-UTR can directly recruit ribosomes or initiation machinery in other systems [122], raising the possibility of a similar mechanism in *T. gondii*.

Furthermore, sustained eIF2α phosphorylation limits ternary complex availability, potentially favoring eIF2-independent initiation pathways, such as those mediated by eIF2D or the DENR-MCTS1 complex in other systems, which deliver initiator tRNA to the ribosome independently of eIF2 and promote reinitiation on uORF-containing transcripts under stress [123]. Systematic interrogation of these pathways will be essential to determine how selective translation in *T. gondii* is maintained as global protein synthesis declines during stage conversion.

### uORF-mediated translational tuning

Upstream open reading frames (uORFs) and upstream AUGs (uAUGs) are extraordinarily prevalent in *T. gondii*, occurring in >90% of transcripts with annotated 5′-UTRs [124,125]. Ribosome profiling and massively parallel reporter assays show that uAUG abundance quantitatively tunes translation efficiency across the parasite transcriptome [124]. Ribosomes frequently assemble at uAUGs but often show limited productive uORF elongation, indicating that these elements act primarily as transient initiation traps that delay scanning and reduce ribosome flux to the main coding sequence [124]. Translation suppression depends largely on uAUG number rather than uORF length or coding potential.

This mechanism explains how differentiation regulators like BFD1 (bottom 2% of translation efficiency) remain transcriptionally poised but translationally repressed until stress [104,124]. ApiAT1 is similarly regulated by a uORF responsive to arginine starvation [126]. However, although uORF-mediated tuning clearly shapes translational efficiency genome-wide, its direct role in controlling bradyzoite differentiation has not been experimentally tested. How 5′-UTR architecture integrates with stress-induced translational reprogramming during stage conversion remains an open question.

### Transcript-level RNA modifications

N^6^-methyladenosine (m^6^A) is the most abundant internal mRNA modification in eukaryotes and regulates multiple aspects of RNA metabolism, including translation [127]. In *T. gondii*, m^6^A is essential for parasite viability and ensures proper mRNA 3′-end formation [128,129]. The core m^6^A writer complex (METTL3, METTL14, WTAP) deposits m^6^A near transcript 3′ ends, where it is recognized by the YTH-domain protein CPSF4 to ensure accurate polyadenylation [129]. Depletion of METTL3 or CPSF4 causes widespread transcriptional readthrough and aberrant transcripts, highlighting a foundational role for m^6^A in shaping the translatable mRNA pool [128,129]. In other eukaryotes, m^6^A also directly modulates translation efficiency [127], raising the possibility that dynamic m^6^A remodeling may contribute to stage-specific translational programs during tachyzoite-to-bradyzoite differentiation.

Recent work further demonstrates that 5′ 7-methylguanosine (m^7^G) capping of mRNA is indispensable in *T. gondii* [130]. The parasite encodes a three-enzyme capping machinery, and depletion of the RNA triphosphatase component leads to widespread loss of capped transcripts, altered gene expression, and complete arrest of parasite replication *in vitro* and *in vivo* [130]. Cap loss preferentially destabilizes short, highly regulated transcripts including histones and invasion factors [130], revealing a strong coupling between RNA modification, translational capacity, and developmental competence.

Beyond mRNA, strain-specific modifications of tRNAs and small RNAs correlate with virulence and cyst-forming potential [131], suggesting broader epitranscriptomic control of protein synthesis. Together, these findings indicate that coordinated RNA modifications, spanning mRNA maturation, stability, and decoding, may reprogram translation during *T. gondii* differentiation and represent a key direction for future research.

### Ribosome heterogeneity and rRNA modifications

Ribosome heterogeneity and rRNA modifications regulate selective translation in many eukaryotes [132,133], yet both remain largely unexplored in *T*. *gondii*. Although strain-specific rRNA variants have been reported [134,135], their functional incorporation into ribosomes and the landscape of rRNA modifications (2′-O-methylation, pseudouridylation) remain unknown.

Notably, the *T. gondii* mitoribosome exhibits extreme structural specialization. Cryo-EM studies reveal that it assembles from over 50 extremely short rRNA molecules [136,137], and that post-transcriptionally added poly(A) tails are incorporated as structural elements within the ribosome, where they are essential for mitoribosome assembly/stability and parasite survival [137]. Nine rRNA sequences are reused, with each copy integrated in different mitoribosome domains [136,137]. Several transcription factor-like proteins are repurposed to compensate for reduced ribosomal domains, including ApiAP2 family members previously considered DNA-binding transcription factors [136,137]. Despite this radical remodeling, conserved catalytic centers are preserved, enabling functional mitochondrial translation [136,137].

While related parasites exhibit developmentally regulated ribosome specialization [138,139], whether cytoplasmic ribosome heterogeneity or dynamic rRNA modification contributes to translational control during *T. gondii* differentiation remains an open and important question.

### Elongation and termination

Although most studies of translational control in *T. gondii* have focused on initiation, elongation may also contribute to developmental reprogramming. Elongation rates shape protein output and co-translational folding. Recent work identified CDPK3 as a stress-activated eEF2 kinase that phosphorylates eEF2 and slows translation when tachyzoites experience prolonged extracellular exposure after host cell egress, particularly at atmospheric oxygen (21% O_2_) [140]. Because bradyzoite differentiation is also triggered by environmental stress, regulated eEF2 activity could represent an additional layer of translational control during stage conversion.

Termination-associated mechanisms may further shape the developmental proteome. Programmed stop-codon readthrough can generate alternative protein isoforms in other systems [141–143]. Furthermore, the NMD pathway regulates the stability of transcripts with aberrant termination codons [144,145]. Defining how elongation dynamics and termination-associated quality control pathways operate in *T. gondii* represents an important frontier for understanding translational reprogramming in differentiation [146].

### Uncharacterized RNA-binding proteins

Only a small fraction of predicted RBPs in the *T. gondii* genome have been functionally investigated. Known regulators like BFD2 and Alba proteins influence translation of differentiation-related transcripts, likely by interacting with cis-elements in 5′- or 3′-UTRs [43,46]. Beyond these examples, the vast majority of RBPs remain unstudied. Given the prevalence of uORF-mediated regulation and the importance of UTRs in translational control, RBPs that recognize specific *cis*-elements within these regions likely represent an expansive but largely uncharacterized layer of post-transcriptional regulation in *T. gondii* stage conversion. Systematic identification of stage-specific RBPs using approaches such as RNA interactome capture or CLIP-seq could uncover new players in translational control during bradyzoite formation.

### Post-translational modifications of translational regulators

In other eukaryotes, post-translational modifications (PTMs) on translation factors and ribosomal proteins, including phosphorylation [147–151], methylation [152,153], ubiquitination [154], and acetylation [155], play critical roles in regulating translation efficiency, factor localization, and protein-protein interactions. In *T. gondii*, eIF2α phosphorylation is the only PTM currently linked to differentiation [93]. eIF1.2 protein levels remain unchanged after 24 hours of alkaline stress despite mediating significant translational changes by this time point [45], raising the possibility that PTMs may regulate its activity. Other initiation factors, ribosomal proteins, and RBPs may similarly undergo dynamic PTMs during stage conversion, though direct evidence remains limited.

Emerging proteomic approaches, such as phosphoproteomics [156,157] and acetylation labeling [158] and ubiquitination profiling [159], could uncover stage-specific PTMs on translation factors and ribosomal proteins. Such modifications may act as molecular switches that rapidly reprogram the translation machinery to favor synthesis of bradyzoite-promoting factors while repressing acute-stage proteins. Identifying these PTMs and their regulatory enzymes may reveal new therapeutic targets for disrupting parasite development and persistence.

## Conclusions and future directions

Translational control has emerged as a central regulatory layer governing *T. gondii* differentiation, enabling rapid and selective proteome remodeling in response to host-imposed stress. Key translation initiation factors, RBPs, transcript-level RNA modifications, alternative splicing, and ncRNAs collectively orchestrate stage-specific protein synthesis that drives bradyzoite formation and persistence.

Despite these advances, multiple layers of translational regulation remain poorly defined. Major open questions include how non-canonical initiation pathways operate during stress, whether ribosome specialization and rRNA modification contribute to selective translation, how mRNA and tRNA modification dynamics shape translational capacity, and how elongation, termination, and RNA surveillance pathways influence developmental gene expression. In parallel, the vast repertoire of uncharacterized RBPs likely represents an additional, largely untapped regulatory layer.

With the advent of high-resolution genomic, ribosome profiling, and structural approaches, the translational landscape of *T. gondii* is now poised for rapid discovery. Defining parasite-specific mechanisms of translational control will not only deepen our understanding of chronic infection biology but may also reveal new therapeutic opportunities to disrupt tissue cyst formation, persistence, and reactivation.

## Open Access

Open access for this article was enabled by the participation of Iowa State University in an all-inclusive Read & Publish agreement with Portland Press and the Biochemical Society.

## Abbreviations

eIF: eukaryotic translation initiation factor
GAP: GTPase-activating protein
lncRNA: long non-coding RNA
ncRNA: non-coding RNA
NMD: nonsense-mediated decay
PTM: post-translational modification
PV: parasitophorous vacuole
RBP: RNA-binding protein
